# Mess. Staines and Everingham, on Inoculation

**Published:** 1808-09

**Authors:** 


### *To the Editors of the Medical and Phyfical Journal.*

GENTLEMEN,

WE shall be much obliged if you will insert in your next Number the following result of a general inoculation for the Small Pox in Wareham, with its effects on those inoculated for Cow Pox.

We are, &c.

STAINES and EVERINGHAM.

*Wareham, August* 10, 1808.

ABOUT

ABOUT the beginning of June, of the present year, a general inoculation for small-pox took place, in consequence of its appearance naturally in several parts of the town; of 283 inoculated, upwards of 30 had it in a dangerous form, of which number 10 died; of these, 8 were under 2 years of age. We are of opinion this unusual fatality was occasioned by the heat of the weather, from the children being kept too warm, and having toast and ale, spirits, &c. given them. Of 81 previously inoculated for cow-pox, 46 were innoculated, many twice, and some three times, for small-pox; of this number 10 had fever and eruptions; we have the particulars of all these cases. In one case there was one eruption, in another two, in most of them less than 20; and in two cases there were upwards of 100 eruptions, preceded by severe fever: in all these cases the eruptions either did not maturate, or maturated prematurely, and all were dry and gone in a week. Of 35 who were not inoculated for small-pox, but who were exposed to it in the severest manner, by living and sleeping together, even when there were deaths in the house from small-pox, not one has taken it; since all these resisted the casual disease, the probable conclusion is, that there would have been no cow-pox failures if none had been subjected to variolous inoculation; even the failures prove the efficacy of cow-pox rendering the subsequent small-pox devoid of danger.

